# The Status Lymphaticus

**Published:** 1907-05-04

**Authors:** 


### The Status Lymphaticus.

A great deal of interest has been aroused during the last few days, both amongst medical men and amongst the laity, by the sudden and unexpected death of Sir Henry Leslie Huntingdon under an anæsthetic. Almost all the daily papers have contained paragraphs upon the subject; we quote the following from the *Daily Chronicle* of Saturday, April 27:—

Medical Mystery.

*Strange Death of Sir Henry Leslie Huntingdon.*

Evidence of the existence of an obscure disease, hitherto little known or studied in this country, was given yesterday at the inquest on Sir Henry Leslie Huntingdon, of the Clock House, Chelsea Embankment.

Sir Henry, who was only 24 years of age and succeeded to the title last Christmas, died suddenly on Wednesday while undergoing an operation for which an anæsthetic had been administered. The post-mortem showed that he suffered from what is known as "status lymphaticus," a disease which Dr. Spilsby, anæsthetist to St. Mary's Hospital, said was a condition that had not till recently engaged attention in this country, but for many years past cases had been recorded in the United States and in Germany.

Those in whom the disease lurked appeared to be liable to die suddenly from trivial causes, and the majority of deaths recorded had taken place under anæsthetics. The disease from which Sir Henry suffered could not, however, have been discovered or suspected from an external examination.

Evidence by Sir Henry's relatives went to show that, to all appearance, he was a healthy man. He was an athlete. Dr. Frankish, the surgeon who was conducting the operation when the patient died, qualified this with the opinion that he was overgrown, being 6 ft. 3½ in. in height. But there was nothing in his condition, so far as the doctor knew, to prevent him taking an anæsthetic.

The jury returned a verdict of death from misadventure.

The medical profession is, as a rule, very sceptical of any condition which is described as a new disease, but the condition known as the "status lymphaticus" is neither new-fangled nor imaginary. There are a considerable number of original papers upon the subject, and it is mentioned and described in the newest text-books of medicine. Much of the investigation that has been done upon the subject has been carried out in Vienna.

The condition may be suspected during life, but unfortunately it cannot yet be diagnosed with certainty until a post-mortem examination is made. The lesions which are found at autopsy may be summarised as being general hyperplasia of all the internal lymphatic tissues of the body. The *Peyer's patches* of the small intestine and the *solitary follicles* of the colon are all enlarged, standing up prominently out of the mucous membrane; the Peyer's patches may be so large that at first sight they may suggest an early stage of typhoid fever, but they may be distinguished from enlargement from this cause by the absence of any inflammatory reaction in or around them; they are simply enlarged, not inflamed; they are sharply demarcated from the surrounding mucosa, and their colour is white or creamy-yellow. The *lymphatic glands* connected with the alimentary canal are also enlarged and pale, without any of the hyperæmia of inflammation; the hyperplasia begins above in the retropharyngeal lymphatic tissue, and affects the tonsils also, so that the patient may seem to be suffering from "tonsils and adenoids"; the lymphatic glands along the œsophagus in the posterior mediastinum, those along the lesser curvature of the stomach, in the portal fissure, and throughout the mesentery are enlarged and pale in the same way, and not infrequently there is a bunch of enlarged lymphatic glands in close contact with the cæcum. Under normal conditions the lymphatic tissues of the mesentery first become recognisable as lymphatic glands at some distance from the attachment of the mesentery to the bowel, but in the "status lymphaticus" the minute collections of lymphoid tissue which are normally present but almost invisible close to where the bowel and the mesentery join are so enlarged as to be recognisable as distinct lymphatic glands, which often stand out like large peas under the mucous membrane. None of the enlarged glands attain any enormous size—the largest are seldom bigger than small hazel nuts, and many are no larger than peas—but they are all considerably larger than the glands in the same position would be in a healthy individual.

The lymphatic glands which are not closely connected with the alimentary canal are less commonly involved in the hyperplasia; those in the neck, axillæ, and groins, for example, may not be affected at all. This is unfortunate, because it is not possible by external examination to tell whether the mesenteric glands are enlarged, and it would be a great boon if we could diagnose the status lymphaticus by palpation of the axillary and inguinal glands. A few cases, however, exhibit hyperplasia of the latter, so that when there is any doubt as to the advisability of giving an anæsthetic to any particular patient, the presence of distinctly palpable glands in axillæ and groins must be an additional contra-indication. The *spleen* is usually enlarged in moderate degree, and its Malpighian bodies are often so prominent that they may be mistaken for tubercles, and lead to a post-mortem diagnosis of general tuberculosis if they are not examined microscopically. Their enlargement is simply a hyperplasia of typical lymphoid tissue, and they show none of the giant cells of tubercle. The spleen, though enlarged and hyperæmic, is soft, and therefore even though it may extend below the costal margin it is very difficult to palpate. This is again unfortunate, for were the enlarged spleen always palpable it would be a great help in arriving at the diagnosis. The enlargement is seldom very great in any case.

The *bone-marrow* of the long bones such as the femur, in which it should be yellow and like fat, is usually still red in the adult as it is in the fœtus. This persistence of the red marrow in the long bones in the status lymphaticus suggests that there is hyperactivity of the blood-forming cells, possibly an attempt to compensate for degenerative blood changes occurring elsewhere in the body. The bone marrow of the femur is similarly red in many conditions of anæmia and cachexia; indeed, it is invariably so in diseases such as pernicious anæmia in which there is a pathological destruction of the blood in the viscera.

The *thymus gland* is persistent in most of the cases of status lymphaticus; it may be very large, not merely covering the pericardium and big vessels in the mediastinum, but even, in some cases, extending upwards into the neck in front of the trachea. It must be remembered, of course, that the mere presence of a large thymus gland does not constitute status lymphaticus, for it normally increases in size up to the end of the second year. At this age it is a gland weighing $\frac{1}{4}$ to $\frac{1}{2}$ oz., consisting of three asymmetrical lobes—a right lower lateral, a left upper lateral, and a superior lobe. It consists of lymphoid tissue arranged in large lobules, and is distinguished from all other varieties of lymphoid tissue by pale concentrically-marked circular areas—Hassall's corpuscles—which are seen in microscopical sections of the gland. After the second year the thymus should undergo rapid degeneration, all the lymphoid tissue disappearing and fat and fibrous tissue taking its place.

There is one well-known disease in which not only persistence, but actual hypertrophy, of the thymus gland almost invariably occurs, and that is Exophthalmic goitre. In this affection the gland may weigh 2 oz. or more even in the adult. In the status lymphaticus the thymus also persists, looks swollen and soft, exudes a milk fluid upon pressure, and may weigh anything between $\frac{1}{2}$ oz. and 2 oz., and measure as much as 4 inches in length. It requires very skilled percussion indeed to detect the slightly-impaired note behind the upper part of the sternum produced by a persistent and enlarged thymus gland; one of the reasons for this being that a resonant note is so easily obtained from the upper part of the sternum by transmission from the lung behind the middle and lower parts of this bone. If, however, in an adult, the physician could determine by percussion that there was enlargement of the thymus gland, the diagnosis of status lymphaticus during life would be less difficult than it is at present.

The changes described above are the only ones which are characteristic of the disease; it has sometimes been stated that the heart and aorta are usually undersized at the same time, that there is nearly always enlargement of the thyroid gland, and that there is frequently coincident rickets; these, however, do not seem to be essential. The main changes characteristic of the disease are in the tonsils, pharynx, mediastinal, and abdominal lymph glands, Peyer's patches, solitary follicles, bone marrow, and thymus gland.

It is difficult to understand quite why these changes should be associated with a tendency to death from apparently trivial causes—for that, unfortunately, is the main symptom of the disease.

The majority of the cases described have been examples of deaths under anæsthetics, and Sir Henry Huntingdon was a "book-case" except in one respect—namely, that by far the greatest number of the patients are children. In regard to the kind of anæsthetic which is most dangerous to them there seems to be little to say; at any rate ether is apparently quite as likely to cause death as is chloroform. There are other things which have killed patients suffering from the status lymphaticus: for example, hypodermic injection of antidiphtheric serum, sudden plunging into cold water, and such minor ailments as simple bronchitis. There are a great many recorded cases of sudden death in children in whom no cause whatever could be assigned, in whom at the post-mortem examination no particular lesion could be found, but in whom the thymus gland has been noted as being very large. Many such cases are probably examples of unrecognised status lymphaticus. The Germans long ago accorded the name "Thymus Tod" to these cases, and "Thymus Tod" and "death from status lymphaticus" are practically synonymous. The following single example will suffice to show how easily such patients may die: a woman was ill in bed, with a skilled nurse in attendance, and in a cot in the same room with the woman was her little daughter, nearly two years old. The child was not very strong, but was not ill, and the nurse, having attended to the mother and having given the child her breakfast of milk from a bottle, left the room to get breakfast for herself; on returning in less than half an hour, she was astounded to find that the child was dead. Nothing had happened to attract the mother's attention; the child had simply and suddenly ceased to live. At the autopsy the thymus was $\frac{3}{4}$ oz. in weight, and there was general enlargement of the intestinal lymph follicles and mesenteric lymphatic glands—the status lymphaticus.

It has been suggested that some cases of sudden death in persons who have fallen into the water, and, though immediately recovered, were dead, and of those who have died suddenly while bathing, are not due to the "cramp" to which they are attributed, but to the status lymphaticus. It is also possible that certain of the sudden deaths that are on record as occurring during convalescence from infectious fevers are due to the same cause. The rôle of the thymus gland in the disease is one upon which all sorts of conjectures are rife. That it may cause stenosis of the trachea in a few cases is possible, and a few deaths may be due to this. Tracheal stenosis, however, will not account for most of the cases, and we are forced to suppose either that there is some obscure nervous reflex from the gland, or else that it has some internal secretion which is in excess in the status lymphaticus. The nervous theory is supported by the well-known condition to which the term "thymic asthma" is applied, and to which laryngismus stridulus and carpopedal tetany are so closely related. On the other hand, there seems to be no reason why these nervous symptoms may not be due to excess of internal secretion.

We have always felt that the rôle of the thymus gland has been too much neglected in relation to the pathology of exophthalmic goître; patients with this complaint are almost as liable as those with the status epilepticus to death from trivial causes, and both conditions are well worthy of further investigation. We hope that the matter will be taken up by the holder of that scholarship at Cambridge, which was founded with the express purpose of advancing our knowledge of the functions of the thymus gland.

